# Multipattern Consensus Regions in Multiple Aligned Protein Sequences and Their Segmentation

**DOI:** 10.1155/BSB/2006/35809

**Published:** 2006-08-13

**Authors:** David KY Chiu, Yan Wang

**Affiliations:** 1 Department of Computing and Information Science, University of Guelph, Guelph, ON, Canada, N1G 2W1

## Abstract

Decomposing a biological sequence into its functional regions is an important prerequisite to understand the molecule. Using the multiple alignments of the sequences, we evaluate a segmentation based on the type of statistical variation pattern from each of the aligned sites. To describe such a more general pattern, we introduce multipattern consensus regions as segmented regions based on conserved as well as interdependent patterns. Thus the proposed consensus region considers patterns that are statistically significant and extends a local neighborhood. To show its relevance in protein sequence analysis, a cancer suppressor gene called p53 is examined. The results show significant associations between the detected regions and tendency of mutations, location on the 3D structure, and cancer hereditable factors that can be inferred from human twin studies.

## 

[1,2,3,4,5,6,7,8,9,10,11,12,13,14,15,16,17,18,19,20,21,22,23,24,25,26,27]
